# Perinatal outcomes of monochorionic diamniotic triplet pregnancies: a case series

**DOI:** 10.1186/s12884-019-2634-7

**Published:** 2019-12-11

**Authors:** Tingting Xu, Xiaodong Wang, Haiyan Yu, Xinghui Liu

**Affiliations:** 1 Department of Obstetrics and Gynecology, West China Second University Hospital, Sichuan University, No. 20, 3rd section, South Renmin Road, Chengdu, 610041 Sichuan China; 2 Key Laboratory of Birth Defects and Related Diseases of Women and Children (Sichuan University), Ministry of Education, Chengdu, China

**Keywords:** Monochorionic diamniotic triplet pregnancies, Perinatal outcomes

## Abstract

**Background:**

Triplet pregnancies are associated with higher fetal morbidity and mortality rates as well as life-threatening maternal complications. Monochorionic diamniotic (MCDA) triplet pregnancies are very rare compared to other types of triplet pregnancies.

**Case presentation:**

We report three cases of MCDA triplet pregnancies between January 2012 and December 2017. Two of these MCDA triplet pregnancies received regular and intensive prenatal care, were diagnosed by ultrasonography during the first trimester or early second trimester, and had good perinatal outcomes. The case with irregular perinatal care had poor outcomes, and the MCDA triplet pregnancy was diagnosed intrapartum.

**Conclusions:**

The possibility of continuing an MCDA triplet pregnancy should be recognized. Early diagnosis, regular antenatal care, close prenatal monitoring, and sufficient communication are recommended to obtain better perinatal outcomes in MCDA triplet pregnancies.

## Background

Triplet pregnancies are associated with higher fetal morbidity and mortality risk such as to be delivered by cesarean at < 29 weeks of gestation and to have > or = 1 infants die as well as life-threatening maternal complications such as preterm premature rupture of membranes, preeclampsia, eclampsia, toxemia, placental abruption, excessive bleeding, to require tocolysis, and postpartum hemorrhage [1, 2]. The incidence of MCDA triplet births is rare compared to other types of triplet pregnancies [3], and the incidence of spontaneous triplet pregnancies is approximately 1/10,000 [4]. However, triplet births have increased due to older maternal age at conception and the increased use of assisted reproductive technology, such as in vitro fertilization (IVF), intracytoplasmic sperm injection (ICSI), gamete intrafallopian transfer (GIFT), and intracervical insemination (ICI) [5]. The proportion of triplet pregnancies conceived using assisted reproductive technology versus naturally has increased in recent years (42.5% vs. 17.7%) [5, 6].

Trichorionic triamniotic (TCTA), dichorionic triamniotic (DCTA), and monochorionic triamniotic (MCTA) triplets are the most common types of triplet pregnancies [7]. Monochorionic diamniotic (MCDA) and dichorionic diamniotic (DCDA) triplet pregnancies are extremely rare. Fennessy et al. [8] reported that 58% (31/53), 32% (17/53), 8% (4/53), and 2% (1/53) of triplet pregnancies are TCTA, DCTA, MCTA, and DCDA, respectively.

Due to special chorionic and amniotic properties in MCDA triplets, cord entanglement between two fetuses can increase the incidence of perinatal morbidity and mortality in MCDA triplets. Nearly ***10*** papers [7, 9–18] related to MCDA triplet pregnancies have been published so far, and the majority have been case reports. However, there is no consensus-based guidance for the perinatal management of MCDA triplet pregnancies. MCDA triplet pregnancies can have good perinatal outcomes with regular prenatal care. Therefore, the antenatal diagnosis, evaluation, and management of MCDA triplet pregnancies are worth further discussion.

We report ***three*** cases of MCDA triplet pregnancies at West China Second University Hospital, a tertiary referral center in west China, between January 2012 and December 2017. In addition, we evaluate the perinatal management and outcomes of these MCDA triplets. In our study, the case with regular and intensive prenatal care had a good perinatal outcome, and the case with irregular perinatal care had poor outcomes.

## Case presentation

### Case 1

**Fig. 1:**
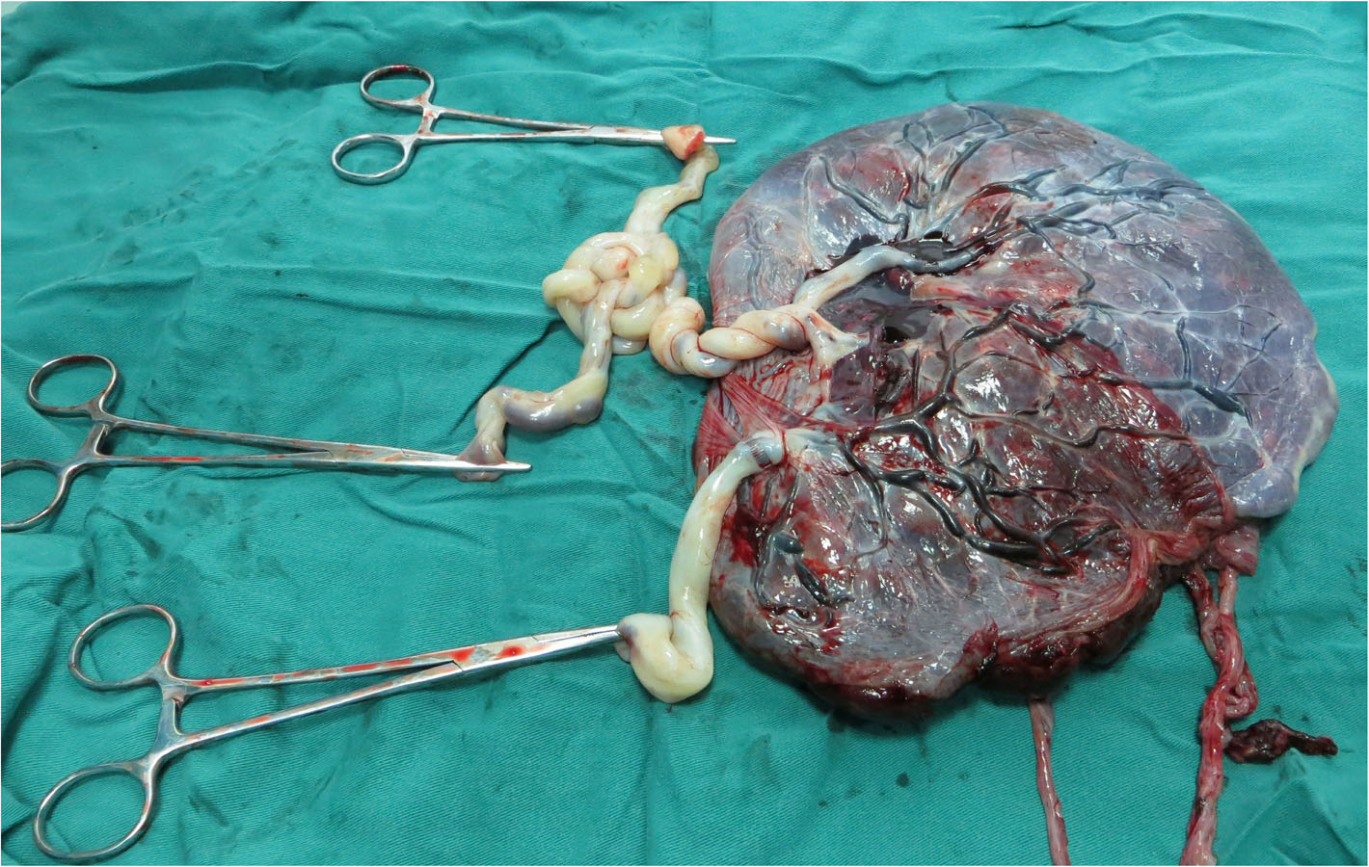
The placental and umbilical cord examination of case 1

A 28-year-old nulliparous woman spontaneously conceived a triplet pregnancy and was transferred to our hospital, where she was diagnosed with MCDA triplets at 13 gestational weeks. Ultrasonography revealed MCDA triplets with two of the triplets (triplets A and B) sharing a single amniotic sac. After extensive counseling regarding the potential risks associated with this condition, the couple opted to continue the triplet pregnancy. Regular and thorough ultrasonography was performed weekly after 28 gestational weeks to monitor fetal weight, bladder capacity, amniotic fluid volume, and Doppler blood flow of the fetal umbilical artery, middle cerebral artery, and ductus venosus (DV). Fetal heart rate monitoring by nonstress tests (NSTs) was carried out weekly after 28 weeks of gestation. The couple refused admission until threatened premature labor at 31 weeks and 3 days of gestation. NSTs were performed twice per day, and ultrasonography was performed twice per week. Antenatal steroids were administered to promote fetal maturation. At 32 + 5 weeks, an emergency cesarean section was performed due to inevitable preterm labor. The diagnosis of an MCDA triple pregnancy was further confirmed after umbilical cord and histopathological examinations (Fig. 1). Cord entanglement in the monoamniotic pair was diagnosed antenatally and confirmed after umbilical cord examination. The birth weights of the three female newborns were triplet A 1510 g, triplet B 1490 g, and triplet C 1350 g. The 1-min Apgar scores of these three surviving infants were 10, 10, and 9. The three neonates were transferred to the neonatal intensive care unit (NICU) and were separately discharged from the hospital after 10, 10 and 12 days. None of the triplets had any neurological abnormalities. The mother and the triplets remained in good health during the two and half years of follow-up.

### Case 2

**Fig. 2:**
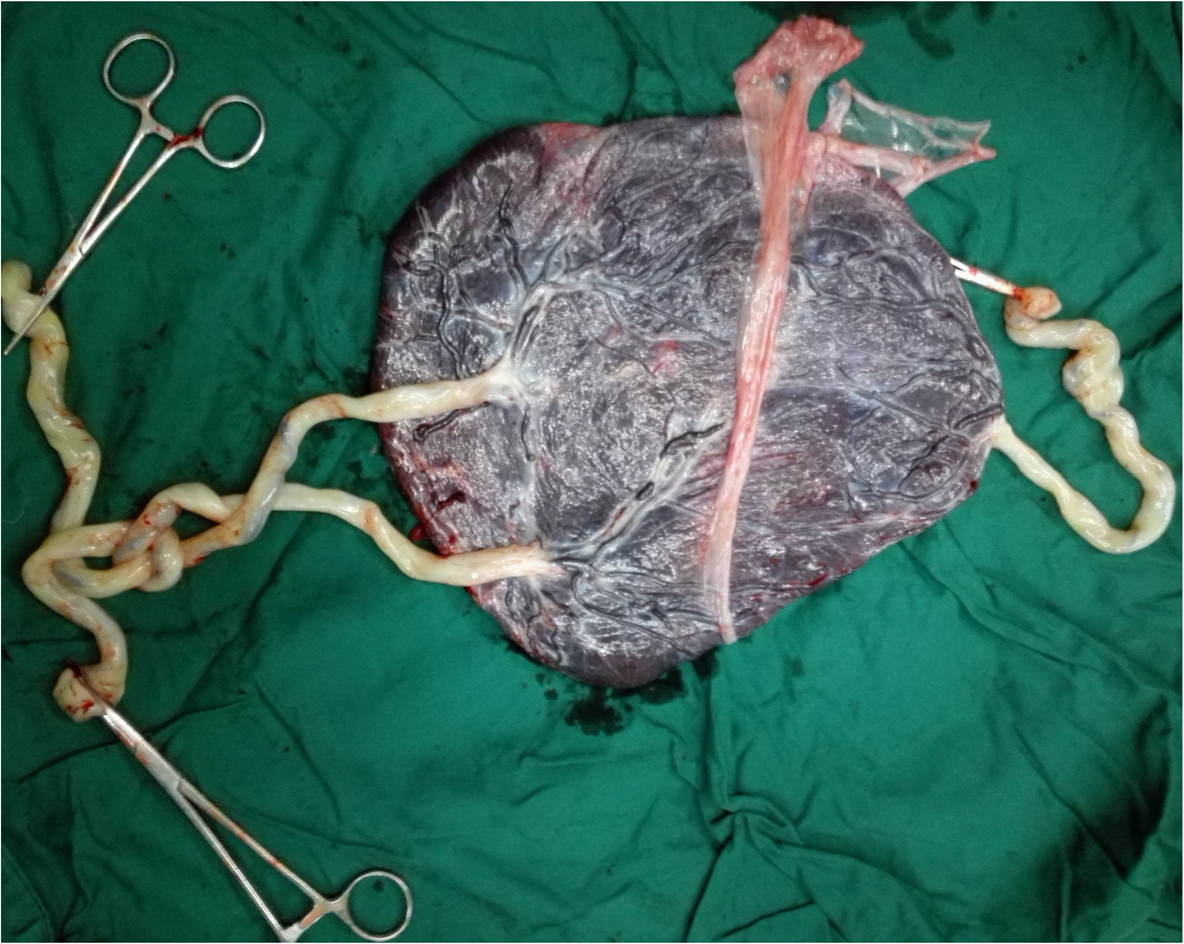
The placental and umbilical cord examination of case 2

A 27-year-old primipara spontaneously conceived a triplet pregnancy and was transferred to our hospital, where she was diagnosed with an MCDA triplet pregnancy at 18 gestational weeks, with triplets B and C sharing a single amniotic sac. The couple opted to continue the pregnancy after extensive counseling. Intensive ultrasonography was performed every week after 28 weeks of gestation. At 29 gestational weeks, the patient was admitted to the hospital. NSTs were performed twice per day, and ultrasonography was performed twice per week. Prophylactic dexamethasone was administered to accelerate fetal lung maturation. At 32 + 3 weeks, an emergency cesarean section was performed due to preterm labor. Umbilical cord entanglement between triplets B and C was diagnosed antenatally and was confirmed during the operation. Histopathological examination confirmed the placental characteristics of the MCDA triplets (Fig. 2). The birth weights of triplets A, B, and C were 1750 g, 1720 g, and 1830 g, respectively, and the 1-min Apgar scores were 10, 10, and 8, respectively. The infants were transferred immediately to the NICU and were discharged after 10, 10 and 19 days. None of the triplets had any neurological abnormalities. The mother and the triplets remained well during the nearly 4 years of follow-up.

### Case 3

A 20-year-old unipara spontaneously conceived a triplet pregnancy and was transferred to our hospital urgently at 28 weeks due to preterm premature rupture of membranes and a serious maternal lung infection. This patient had received no regular care during pregnancy. Antenatal corticosteroids were administered to stimulate fetal lung maturation. An emergency cesarean section was performed at 28 + 3 weeks due to maternal acute heart failure complicated with severe pneumonia. Three male newborns were delivered, with triplets A and B sharing an amniotic sac weighing 1000 g and 900 g, and triplet C in another amniotic sac weighing 990 g. The diagnosis of an MCDA triplet pregnancy was established by the placenta and umbilical cord examinations and histopathological examination. The 1-min Apgar scores of these three infants were 5, 7, and 7. The neonates were immediately transferred to the NICU. Triplets A and B died 23 days after birth, and triplet C died after 6 days due to multiple organ failure.

## Discussion

Triplet pregnancies are associated with an increased risk of maternal complications, including gestational diabetes mellitus, gestational hypertension, preeclampsia, eclampsia, toxemia, intrahepatic cholestasis of pregnancy, anemia, preterm premature rupture of membranes, placental abruption, excessive bleeding, to require tocolysis, postpartum hemorrhage, and abortion [1, 19–21].

Such pregnancies are also related to poorer neonatal outcomes, such as preterm birth (delivered by cesarean at < 29 weeks of gestation), intrauterine growth restriction, low birth weight, congenital anomalies, fetal death, and to have > or = 1 infants die, neurological disability and other short-and long-term disabilities [1, 8, 22]. Besides, the fetal loss rate of triplets is nearly 25% [23].

The prevalence rate of triplet gestations in the United States and Canada has been approximately 154 and 84 per 100,000 live births, respectively, in the last few years [24]. Moreover, at our hospital, the prevalence rate of triplets was 11.14/10,000, and the proportion of MCDA triplet pregnancies was 4.41% (3/68) between January 2012 and December 2017.

The placentation of monochorionic is characterized by placental vascular anastomoses and thus inter fetal transfusion [23]. Chorionicity contributes greatly to adverse perinatal outcomes, and monochorionicity is associated with a higher rate of perinatal complications [5]. The rates of fetal death after 22 weeks and neonatal death in MCTA, DCTA, and TCTA triplet pregnancies are 5.3, 3.2, and 2.1%, respectively [25]. In Japan, the perinatal mortality rates of monochorionic, dichorionic, trichorionic triplet deliveries are 12.5, 4.4, and 2.0%, respectively [26]. The incidence of preterm birth before 32 weeks in triplet pregnancies is 24.1-fold higher than that in singleton pregnancies and 3.3-fold higher than that in twin pregnancies [27]. Nearly 14% of triplets are delivered before 30 weeks, and 61% of triplets are born before 34 weeks [26]. Compared with singleton pregnancies, triplet pregnancies are nearly four times more expensive [27]. Therefore, triplet pregnancies are not recommended due to higher fetal morbidity and mortality.

When a triplet pregnancy is diagnosed, management options, including continuing the pregnancy with expectant treatment or elective reduction to twins or a singleton, should be discussed with the couple [28, 29]. Elective reduction to twins or a singleton requires occlusion of the umbilical vessel by laser photocoagulation, bipolar electrocoagulation and radiofrequency ablation [30].

When a single embryo splits between 4 and 8 days after fertilization, an MCDA twin pregnancy occurs, and MCTA triplets occur if one of the twins further splits before the 8th day after fertilization [31]. MCDA triplets are extremely rare and can be diagnosed by ultrasonography. Cord entanglement between two fetuses can increase the incidence of perinatal morbidity and mortality in MCDA triplets. Due to the rarity of MCDA triplet pregnancies, there is no standard management protocol.

To the best of our knowledge, there have been nearly ***10*** papers [7, 9–18] related to MCDA triplet pregnancies published in English, and the results are summarized in Table 1.

**Table 1:** The character of the included study

Study ID	Maternal age (years)	Gravida	Para	Spontaneously conceived	Diagnose weeks	Gestational age (weeks)	Delivery method	Maternal complication	Pregnancy outcomes	Umbilical cord entanglement
Sepulveda 2003 [14]	41	3	2	not mention	13	28	CS	No	Selective feticide of the conjoined twins was conducted at 16 gestational weeks, the normal triplet died at 28 weeks weighing 1010 g	Yes
Sepulveda 2009 [15]	33	3	1	not mention	17	31	CS	No	Spontaneous cessation of blood flow to the acardiac fetus at 23 weeks, another two fetuses were alive and well.	Yes
Yong 2010 [11]	32	1	0	Yes	25–26	30	CS	No	Three healthy triplets	Yes
Youssef 2012 [9]	22	1	0	Yes	11	Termination of pregnancy		No	/	not mention
Sellami 2013 [18]	20	1	0	Yes	21	Termination of pregnancy		No	/	No
Talebian 2015 [16]	38	1	0	No (ART)	12 + 3	17	VD	No	selective feticide of the conjoined fetuses, then the membrane ruptured the day after the feticide and inevitable abortion	No
Yonetani 2015 [7]	34	1	0	Yes	12	35	CS	No	Three healthy newborns	Yes
Suizu 2016 [10]	34	1	0	No (ART)	8 + 2	33	CS	decreased platelet count	Three healthy and survival infants	Yes
Anglim 2016 [17]	41	1	0	No (ART)	25	30 + 5	CS	severe hyponatraemia associated with pre-eclampsia	Three healthy triplets	not mention
May 2016 [12]	30	2	1	not mention	Histology of the placenta	27 + 6	CS	No	A live pump twin and two conjoined acardiac TRAP recipients	No
S Bari. 2018 [13]	25	1	0	not mention	34+	34+	CS	respiratory distress	One alive male fetus and two female dead fetus	No
Our cases										
No. 1	28	3	0	Yes	13 + 4	32 + 5	CS	No	Three healthy and survival newborns	Yes
No. 2	27	3	0	Yes	18 + 5	32 + 3	CS	No	Three healthy and survival infants	Yes
No. 3	20	2	0	Yes	12+	28 + 3	CS	lung infection and heart failure	Three survival male babies, the parents give up further treatment	Yes

*CS* Cesarean section, *VD* Vaginal delivery, *TRAP* Twin reversed arterial perfusion, *ART* Assisted reproductive technology

Most of the cases were diagnosed by ultrasound in the first trimester or early second trimester, especially in Suizu's report [10], where MCDA triplet pregnancies were diagnosed around 9 weeks. Four cases of conjoined twins in MCDA triplet pregnancies have been reported [12, 14, 16, 18]. In May’s study, the MCDA triplets consisted of two conjoined acardiac twins and one surviving fetus with a normal heart. The live newborn was delivered by cesarean section at 27 + 6 weeks and was discharged from the NICU after a 14.5-week stay [12]. Two cases were reported of MCDA triplets in which the conjoined twins underwent selective feticide at 16 gestational weeks; however, in one case, intrauterine demise of the normal triplet occurred at 28 weeks [14], and in the other case, the membranes ruptured the day after the procedure, resulting in an inevitable abortion [16]. Sellami’s case report [18] describes xipho-omphalopagus conjoined twins in an MCDA triplet pregnancy that was terminated at 21 weeks. Sepulveda [15] reported an acardiac fetus complicating an MCDA triplet pregnancy. The blood flow of the acardiac fetus spontaneously ceased at 23 weeks, while the other two fetuses remained alive and well. Four cases of MCDA triplet pregnancies with regular prenatal care had good perinatal outcomes. These 12 healthy infants remained healthy without any other major complications during follow-up after birth [7, 10, 11, 17]. One case with no regular antenatal care was misdiagnosed as a twin pregnancy, and MCDA triplets were established during the cesarean section; one live fetus and two dead fetuses were delivered at 34+ gestational weeks [13]. Detailed information on these cases is provided in Table 1.

In our study, the cases with careful and regular prenatal care had good perinatal outcomes, and six healthy babies remained well without any short-or long-term abnormalities. Conversely, the case (case 3) with no regular antenatal care during pregnancy had a poor outcome.

It is true that some of the conditions discussed from cited papers couldn’t be prevented even with regular prenatal care. However, from this data in Table 1, we can see most of the cases were diagnosed by ultrasound in the first trimester or early second trimester and conjoined twins were common in MCDA triplets. Besides, we also can see that for most MCDA triplet pregnancies with regular prenatal care had good perinatal outcomes expect some uncontrollable and unavoidable factors.

## Conclusion

Taking together the above-published articles and our cases, we conclude that intensive prenatal care by a multidisciplinary team is important for obtaining a better perinatal outcome. In addition, umbilical cord entanglement between two fetuses sharing the same amniotic sac was found in most of our MCDA triplets, which can increase the incidence of perinatal morbidity and mortality. MCDA triplet pregnancies with cord entanglement can be successfully managed by early diagnosis, regular antenatal care, close prenatal monitoring, sufficient communication, and elective delivery.

Therefore, it is very important for women with an MCDA triplet pregnancy to obtain intensive prenatal care, which can help obstetricians adjust perinatal evaluations and management strategies in a timely manner to avoid adverse perinatal complications. Weekly ultrasounds are recommended after 28 gestational weeks. It is better for women with MCDA triplet pregnancies to be admitted to the hospital at 28–30 weeks and complete fetal lung maturation. A histopathological examination of the placenta to confirm the chorionicity and amnionicity of an MCDA triplet pregnancy is necessary after delivery.

## Data Availability

The data set supporting the conclusions of this article is included in the article.

## Abbreviations

CS: Cesarean section
DCDA: Dichorionic diamniotic
DCTA: dichorionic triamniotic
DV: Ductus venosus
MCDA: Monochorionic diamniotic
MCTA: Monochorionic triamniotic
NICU: Neonatal intensive care unit
NST: Nonstress testing
TCTA: Trichorionic triamniotic
TRAP: Twin reversed arterial perfusion
